# Population structure of *Anopheles (Kerteszia) bellator* in the Brazilian Atlantic Forest

**DOI:** 10.1590/0074-02760240287

**Published:** 2025-07-21

**Authors:** Iara Carolini Pinheiro, Kamila Voges, Andre Akira Gonzaga Yoshikawa, Sabrina Fernandes Cardoso, Antonio Bernardo Carvalho, André Nóbrega Pitaluga, Luísa Damazio Pitaluga Rona

**Affiliations:** 1 Universidade Federal de Santa Catarina, Departamento de Biologia Celular, Embriologia e Genética, Florianópolis, SC, Brasil; 2 Secretaria de Estado da Saúde de Santa Catarina, Diretoria de Vigilância Epidemiológica, Florianópolis, SC, Brasil; 3 Universidade Federal do Rio de Janeiro, Departamento de Genética, Rio de Janeiro, RJ, Brasil; 4 Fundação Oswaldo Cruz-Fiocruz, Instituto Oswaldo Cruz, Rio de Janeiro, RJ, Brasil; 5 Conselho Nacional de Desenvolvimento Científico e Tecnológico, Instituto Nacional de Ciência, Tecnologia e Inovação em Entomologia Molecular, Rio de Janeiro, RJ, Brasil

**Keywords:** Anopheles (Kerteszia) Bellator, *cytochrome C oxidase I* (*COI*), bromeliad malaria, cryptic species

## Abstract

**BACKGROUND:**

Malaria, caused by protozoa of the genus *Plasmodium* and transmitted by *Anopheles* mosquitoes, remains a significant global health concern. In 2022, approximately 249 million malaria cases were reported worldwide, including 163,000 in Brazil. In the Atlantic Forest, *An. bellator* and *An. cruzii* are the primary vectors of malaria transmission.

**OBJECTIVES:**

This study used a *cytochrome C oxidase I* (*COI*) gene fragment to investigate the genetic population structure of *An. bellator* in the Brazilian Atlantic Forest.

**METHODS:**

Mosquitoes were collected from Itaparica (BA), Camacan (BA), Ilha Grande (RJ), Antonina (PR), Ilha do Mel (PR), and Florianópolis (SC). They were morphologically identified and individually photographed. DNA was extracted, and a *COI* gene fragment was amplified using polymerase chain reaction (PCR), purified, and sequenced. Additionally, sequences from Trinidad, Colombia, and São Paulo State, obtained from GenBank, were included in the analysis. These sequences were used for molecular identification, genetic variation analysis within and between populations, and phylogenetic assessment.

**FINDINGS:**

The analysis revealed that the *An. bellator* population from Trinidad is genetically distinct from all analysed populations. Furthermore, the Camacan population forms a distinct group separate from the Itaparica population, with both differing from the southern Brazilian populations and that of Colombia. Additionally, the data suggest that the southern Brazilian populations may represent distinct incipient species, particularly the Ilha Grande sample. This divergence is strongly supported by fixed genetic differences, high *F*_*ST*_ values, and genealogical analysis.

**MAIN CONCLUSION:**

The findings provide strong evidence of cryptic species within *An. bellator*, which appears to consist of at least three sibling groups: one from Trinidad and Tobago; *An. bellator* B, which includes sequences from Camacan; and *An. bellator* A, which contains sequences from Colombia, Itaparica, Ilha Grande, São Paulo, Florianópolis, Ilha do Mel, and Antonina. Despite its geographical proximity to Camacan (280 km), the Itaparica population clusters with southern populations ~2,000 km away, while remaining genetically distinct from them. Additionally, the study identified higher *F*_*ST*_ values between the Ilha Grande population and other southern Brazilian samples, highlighting further genetic divergence.

Malaria is a tropical disease caused by *Plasmodium* parasites, transmitted through the bites of female *Anopheles* mosquitoes.^1^ In the 1950s, the World Health Organization (WHO) and other international institutions committed to eradicating malaria.^2^ However, for malaria control and prevention programs to be effective, it is essential to accurately detect and identify vector species. This task can be challenging due to the presence of cryptic species complexes within the *Anopheles* genus,^3^ where different sibling species may play distinct roles in malaria transmission, as seen in the *Anopheles gambiae* complex in Africa.^4^

*Anopheles* (*Kerteszia*) *bellator* is a relevant malaria vector in the Brazilian Atlantic Forest.^5^ This bromeliad-breeding mosquito is distributed along the coast of Rio Grande do Sul in southern Brazil to Sergipe in the northeast and reappears in eastern Venezuela, indicating a discontinuous distribution.^5^ ^,^ ^6^ Between 1930 and 1960, *An. bellator*, along with *Anopheles* (*Kerteszia*) *cruzii* and *Anopheles* (*Kerteszia*) *homunculus*, was identified as one of the primary malaria vectors when the disease was endemic in southern Brazil.^7^ Similarly, on Trinidad Island (Trinidad & Tobago), *An. bellator* became the primary vector of bromeliad malaria in the late 1930s, leading to a severe public health crisis with high mortality rates.^8^ ^,^ ^9^

Previous studies have identified significant genetic differences among *An. bellator* populations in the Atlantic Forest, particularly when comparing populations from Bahia (BA) with those from the Southern and Southeastern regions of Brazil. Isoenzyme analyses revealed that the *An. bellator* population from Trinidad Island (Trinidad & Tobago) is genetically distinct from Brazilian populations in Florianópolis (Santa Catarina - SC), Cananéia (São Paulo - SP), and Itaparica (BA), suggesting a potential early stage of speciation. Additionally, among the three Brazilian samples, Florianópolis and Cananéia exhibited genetic proximity, while showing considerable divergence from Itaparica.^10^ Subsequent analyses using *timeless* and *Clock* genes as molecular markers supported the isoenzyme findings, showing that another population from Bahia State (Camacan, located 280 km south of Itaparica) is also genetically distinct from those in southern and southeastern Brazil, including Ilha Grande (Rio de Janeiro - RJ), Cananéia (SP), and Ilha do Mel (Paraná - PR).^11^ These findings raise important questions: Does *An. bellator* population in Camacan belong to the same group as that in Itaparica? Are there additional species in the *An. bellator* complex? To address these questions and to better understand the genetic variability and divergence among *An. bellator* populations, a fragment of the *cytochrome C oxidase I* (*COI*) gene was analysed in *An. bellator* populations from southern, southeastern, and northeastern Brazil, as well as from Trinidad, and Colombia.

## MATERIALS AND METHODS

*Mosquito collection and morphological identification* - The mosquitoes used in this study were collected from various locations within the Brazilian Atlantic Forest, including Florianópolis - Ilha do Arvoredo (SC), Ilha do Mel (PR), Antonina (PR), Ilha Grande (RJ), Camacan (BA), and Itaparica (BA). Of the 38 specimens analysed, DNA from eight was kindly provided by Voges et al.,^11^ who focused on the nuclear genes *timeless* and *Clock* [Supplementary data (Table I)]. The remaining 30 *An*. *bellator* specimens were collected specifically for this study. Both adult and immature stages were sampled, processed, and preserved by the methods described by Dias et al.^12^ Specimens were identified morphologically following Consoli & Lourenço-de-Oliveira.^13^ Each mosquito was individually photographed, highlighting key morphological traits used to determine its genus, subgenus, and species. Detailed information on field collections of each specimen is provided in Supplementary data (Table I).

*Molecular analysis* - For the molecular analysis, 38 mosquitoes were used. DNA from eight specimens was kindly provided by Voges et al.^11^ [Supplementary data (Table I)]. For the remaining 30 insects collected for this study, genomic DNA was extracted from each mosquito using a non-destructive enzymatic method as described by Santos et al.,^14^ with reagents from the Puregene Core kit A (Qiagen). This method preserves key morphological features, such as the exoskeleton and male genitalia. Mosquitoes were initially stored in 100% ethanol at -20ºC. They were placed in tubes containing 100 µL of lysis buffer and 1 µL of proteinase K (20 mg/mL). After incubation for three days at 45ºC, followed by 1 min on ice, 33 µL of precipitation buffer was added, mixed by inversion, and incubated on ice for 5 min. The samples were then centrifuged for 3 min at 21,130 rcf, and the supernatant was transferred to a new tube. RNase (0.5 µL, 4 µg/mL) was added, followed by three incubation steps: 65ºC for 15 min, 37ºC for 15 min, and 65ºC for 15 min with 2 µL of proteinase K. The samples were placed on ice for 1 min, then 33 µL of precipitation buffer was added again, mixed by inversion, and kept on ice for another 5 min. After centrifugation at 21,130 rcf for 3 min, the supernatant was transferred to a new tube containing 1 µL of Invitrogen™ GlycoBlue™ Coprecipitant (15 mg/mL), mixed, and then 100 µL of absolute isopropanol was added and homogenised by inversion. The samples were centrifuged at 21,130 rcf for 5 min, the supernatant was discarded, and the resulting blue pellets were briefly air-dried at room temperature. Each pellet was then washed with 100 µL of 70% ethanol, centrifuged at 21,130 rcf for 1 min, and the supernatant was discarded. The pellets were left to air dry for approximately 10 min. DNA was dissolved in 50 µL of DNA hydration solution, incubated at 65ºC for 1 h, and left overnight at room temperature. DNA concentration was measured using Qubit with dsDNA Quantitation high sensitivity reagents (Invitrogen). The tubes were then stored at -20ºC.

To isolate *COI* gene fragments, a pair of degenerated primers was specifically designed for the *Kerteszia* subgenus, based on the primers described by Kumar et al.^15^ These primers, named KERT1-F (5’ GAG GAT TYG GAA ATT GAT TAG TTC C 3’) and KERT1-R (5’ AAA AAT YTT AAT TCC TGT TGG YAC AGC 3’), were used in polymerase chain reaction (PCR) with *An. bellator* genomic DNA. PCR was performed using a GoTaq G2 Hot Start Taq Polymerase (Promega) on an Applied Biosystems^®^ thermocycler under the following conditions: one cycle at 95ºC for 9 min, followed by 40 cycles at 95ºC for 30 s, 47ºC for 45 s, and 62ºC for 45 s, with a final extension cycle of 7 min at 72ºC. PCR products were visualised on a 1% agarose gel. Positive amplicons were purified using the Wizard SV Gel and PCR Clean-Up System kit (Promega). Sequencing of the purified amplicons was carried out in both directions on an ABI Prism 3730 DNA sequencer at the Oswaldo Cruz Institute, using the ABI Prism Big Dye Terminator Cycle Sequencing Ready Reaction kit (Applied Biosystems, Foster City, USA).

*DNA sequences analysis* - Sequence quality was verified using CHROMAS version 2.4, and consensus sequences were assembled in SeqMan version 7.0. Molecular identification based on these sequences was conducted using the National Centre for Biotechnology Information (NCBI) (BLAST: Basic Local Alignment Search Tool) database. DNA sequences were aligned with Clustal X,^16^ and a phylogenetic tree for the *COI* gene was constructed using the Maximum Likelihood method in IQ-Tree version 2.1.2,^17^ with the best-fit substitution model. The resulting IQTREE file was then uploaded to the iTOL (Interactive Tree of Life)^18^ platform for visualisation.

Intra-population metrics, including the number of polymorphic sites (*S*), nucleotide diversity based on the average number of pairwise differences (*π*), nucleotide diversity based on the total number of mutations (*θ*), and Tajima’s neutrality test (*D*_*T*_ ),^19^ were obtained for each population using DNA Sequence Polymorphism version 6.12.06.^20^ Inter-population metrics were also calculated for each pairwise comparison using the same software, including the population differentiation coefficient (*F*_*ST*_ ), the average number of nucleotide substitutions per site (*D*_*XY*_ ), the number of net nucleotide substitutions per site (*D*_*a*_ ), the number of shared polymorphisms (*S*_*S*_ ), the number of fixed differences (*S*_*F*_ ) between populations, and the number of polymorphisms exclusive to each population (*S*_*1*_ and *S*_*2*_ ). The haplotype network was generated using PopArt 1.7^21^ and DNA Sequence Polymorphism version 6.12.06.^20^

## RESULTS AND DISCUSSION

*Molecular data and genealogies* - A total of 38 *COI* gene sequences were obtained in this study, including eight sequences from Florianópolis (SC), three from Ilha do Mel (PR), one (1) from Antonina (PR), 11 from Ilha Grande (RJ), five from Camacan (BA), and 10 from Itaparica (BA). These sequences have been submitted to GenBank (GenBank accession numbers: PP422261 - PP422298) [Supplementary data (Table I)]. Based on molecular identification using the NCBI database, the sequences from Florianópolis, Ilha do Mel, and Antonina showed 99-100% similarity to *An. b*e*llator*. Sequences from Itaparica showed 97% similarity, while the lowest similarity values, around 94%, were found in sequences from Camacan. According to Hebert et al.,^22^ genetic divergence between species is usually over 3%, suggesting that the Camacan sequences likely belong to a different species within the *An. bellator* complex. Given that the sequences from the NCBI database originate from São Paulo State,^23^ ^,^ ^24^ it is expected that samples from Florianópolis, Ilha do Mel, and Antonina showed the highest similarity to them. This is consistent with the findings of Voges et al.^11^ and Carvalho-Pinto & Lourenço-de-Oliveira,^10^ which demonstrated that sequences from southern and southeastern Brazil are more closely related to each other than to those from Camacan and Itaparica.

**Fig. 1:**
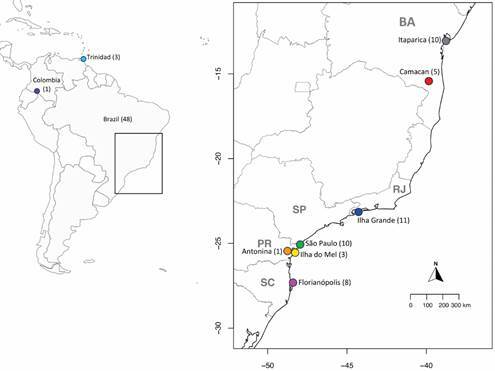
collection sites of *Anopheles bellator* populations. On the left, a map of South America highlights the samples from Colombia and Trinidad, marked with coloured circles. On the right, a zoomed-in view of the boxed area on the Brazil map highlights the specific sample collection sites (also marked with coloured circles). The x and y axes on this map represent longitude and latitude, respectively. The number of samples per locality is indicated in parentheses. Samples from Trinidad, Colombia, and São Paulo State were obtained from GenBank (accession numbers: OQ272307 - OQ272319 and KU551287). The maps were created using the *sf*, *maps*, *mapdata*, and *rworldmap* packages^31^ ^,^ ^32^ ^,^ ^33^ ^,^ ^34^ ^,^ ^35^ in R Software, version 4.3.1.^36^

The genealogical relationships among haplotypes for the *COI* gene were inferred using Maximum Likelihood analysis. In addition to the 38 sequences obtained in this study, three *COI* sequences from Trinidad, one (1) from Colombia, and 10 from São Paulo State, obtained from GenBank (accession numbers: OQ272307 - OQ272319 and KU551287), were included in the analysis (Fig. 1).

**Fig. 2:**
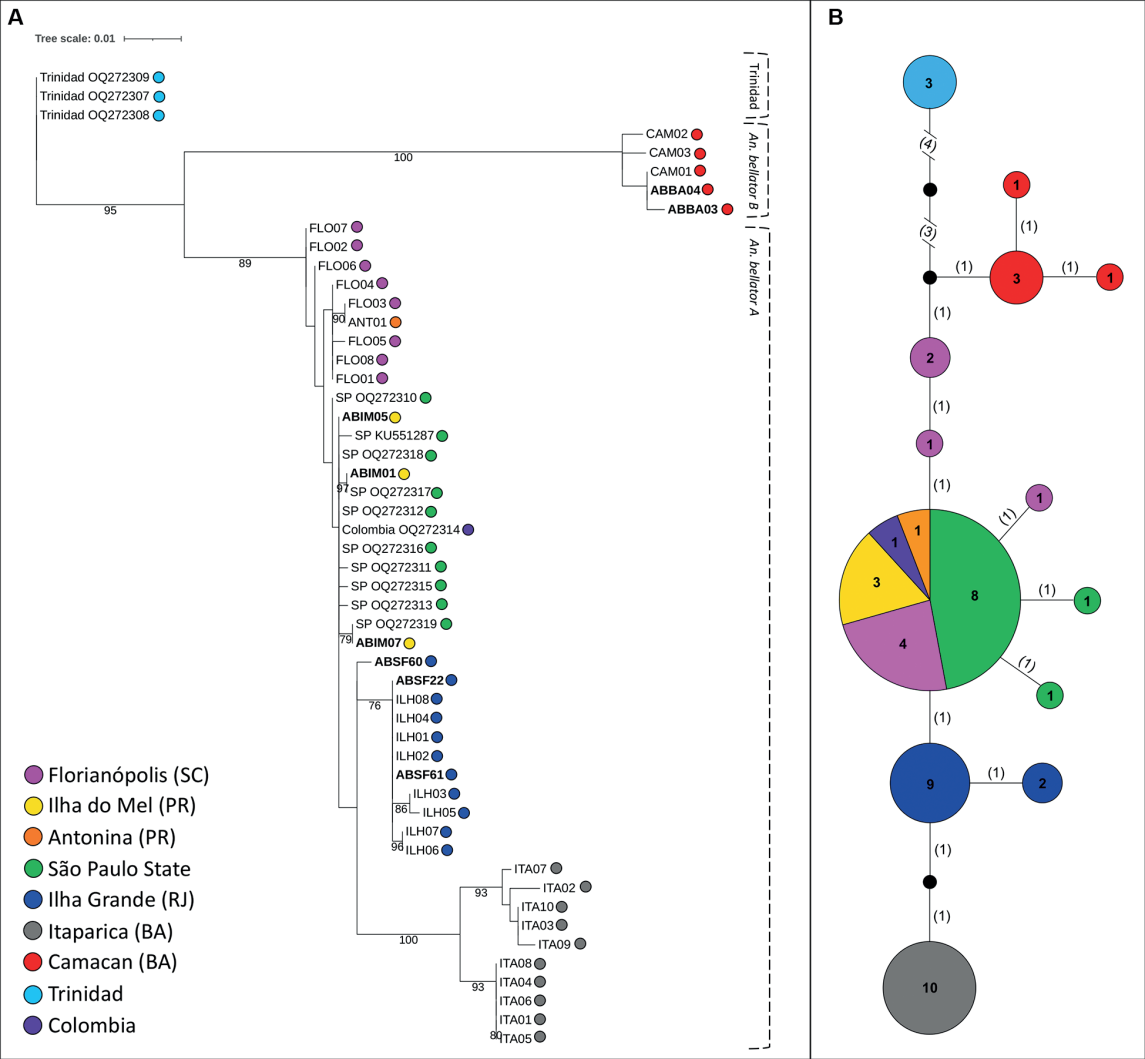
*Anopheles bellator* genealogies. (A) Maximum likelihood tree of *An. bellator COI* sequences using the HKY+I model. This phylogenetic analysis suggests that *An. bellator* comprises at least three cryptic species. One group originates from Trinidad and Tobago, a second group, *An. bellator* B, includes samples from Camacan, and a third group, *An. bellator* A, is found in Colombia, Itaparica, and other southern Brazilian regions. Notably, the haplotypes of the latter group do not appear randomly in this tree, indicating two possible incipient species within *An. bellator* A: (i) from Itaparica and (ii) from Ilha Grande. Southern Brazilian populations (São Paulo, Florianópolis, Ilha do Mel and Antonina) and Colombia showed no differentiation. Node values represent bootstrap percentages based on 1,000 replications, with only values above 75% displayed. The codes next to the São Paulo State, Trinidad, and Colombia samples are the GenBank accession numbers. The sequence IDs highlighted in bold represent samples kindly provided by Voges et al.^11^ SC: Santa Catarina State; PR: Paraná State; SP: São Paulo State; RJ: Rio de Janeiro State; BA: Bahia State. (B) Haplotype network of mitochondrial *COI* gene sequences for *An. bellator* populations. This haplotype network aligns with the ML tree, indicating cryptic speciation within *An. bellator*. The size of each circle reflects the frequency of the corresponding haplotype, with the numbers inside the circles indicating the number of sequences comprising each haplotype. The numbers in parentheses represent the mutational steps separating the haplotypes. Small black circles indicate missing intermediate haplotypes.

This analysis found that the *An. bellator* population from Trinidad (Trinidad & Tobago) is genetically distinct from all Brazilian populations and from Colombia, supporting findings from a previous isoenzyme study.^10^ The *An*. *bellator* haplotypes from Brazil and Colombia were divided into two groups (henceforth referred to as *An. bellator* A and B, based on Voges et al.^11^ findings): *An. bellator* B consisted solely of sequences from Camacan and *An. bellator* A included sequences from Colombia and populations from the south, southeast, and Itaparica, with the Itaparica sequences clustering separately (Fig. 2A). The tree showed no clear separation between the Brazilian sequences from the Southern and Southeastern regions and the sequence from Colombia, though some differentiation was evident, particularly for Ilha Grande.

A network of genealogical relationships among *An. bellator* haplotypes were also estimated (Fig. 2B), supporting the ML tree and showing that *An. bellator* consists of at least three sibling groups: Trinidad, *An. bellator* A and *An. bellator* B. Preliminary evidence also suggests the possibility of two additional distinct incipient species within *An. bellator* A: (i) those from Itaparica and (ii) Ilha Grande. The sequences from other southern Brazilian populations (Florianópolis, Ilha do Mel, Antonina, and São Paulo) and the sequence from Colombia showed no differentiation, as they shared haplotypes.

In both analyses, the ML tree and the haplotype network, the sequences from Ilha do Mel and Antonina cluster together. With only four sequences in total, they will be referred to collectively as ‘Paraná’ from now on to simplify the analysis.

*Intra-population diversity and genetic divergence between An. bellator populations* - Supplementary data (Table II) shows the number of DNA sequences analysed for each *An. bellator* population (*n*). Based on these sequences, the number of polymorphic sites (*S*), as well as the π and θ values, were calculated for each population. The Itaparica (BA) population was the most polymorphic, displaying the highest values of *π* and θ, as well as the greatest number of polymorphic sites (*S*). In contrast, the Ilha Grande (RJ) population was one of the least polymorphic, with the lowest *π* and *θ* values. Supplementary data (Table II) also shows the results of Tajima’s *D* test, which were non-significant in all cases (p > 0.10), indicating no deviations from neutrality.

Table presents the pairwise *F*_*ST*_ estimates for population differentiation between all *An. bellator* populations, along with their geographic distances. Most pairwise comparisons showed high *F*_*ST*_ values (*F*_*ST*_ > 0.7), except for the ^*^Paraná comparisons, where *F*_*ST*_ < 0.1. Table also shows the *D*_*XY*_ and *D*_*a*_ values for differentiation, which follow the same pattern as the *F*_*ST*_ estimates. Additionally, it includes the distribution of four categories of segregating sites for each comparison: the number of shared polymorphisms (*S*_*S*_ ) and the number of fixed differences (*S*_*F*_ ) between the two populations, and the number of polymorphisms exclusive to each sample (*S*_*1*_ and *S*_*1*_ ).

**TABLE:** Genetic differentiation among all populations of *Anopheles bellator*

Populations	*F*_*ST*_	Km	*D*_*XY*_	*Da*	*S*_*S*_	*S*_*F*_	*S*_*1*_	*S*_*2*_
Trinidad x São Paulo	0.98268	4,185	0.03990	0.03921	0	22	0	4
Trinidad x Ilha Grande	0.97829	4,153	0.04016	0.03929	0	10	0	2
Trinidad x ^*^Paraná	0.97561	4,220	0.03973	0.03876	0	10	0	1
Trinidad x Florianópolis	0.95846	4,450	0.04257	0.04080	0	16	0	4
Trinidad x Camacan	0.94167	3,735	0.05063	0.04768	0	11	0	3
Trinidad x Itaparica	0.90842	3,600	0.05156	0.04684	0	14	0	7
Camacan x ^*^Paraná	0.96000	1,447	0.05348	0.05134	0	9	2	0
Camacan x Ilha Grande	0.95231	993	0.05348	0.05093	0	10	2	1
Camacan x São Paulo	0.94565	1,380	0.05232	0.04948	1	10	1	1
Camacan x Itaparica	0.92648	280	0.05612	0.05199	0	12	3	1
Camacan x Florianópolis	0.89945	1,632	0.05485	0.04934	1	11	2	2
Itaparica x ^*^Paraná	0.82112	1,710	0.02733	0.02244	0	4	4	1
Itaparica x São Paulo	0.77778	1,640	0.02593	0.02016	0	4	7	2
Itaparica x Florianópolis	0.73144	1,900	0.02545	0.01861	0	4	8	4
Itaparica x Ilha Grande	0.71609	1,265	0.01862	0.01333	1	3	4	1
Ilha Grande x São Paulo	0.86188	442	0.01167	0.01006	0	2	2	2
Ilha Grande x ^*^Paraná	0.82240	483	0.01101	0.00906	0	1	2	1
Ilha Grande x Florianópolis	0.72435	642	0.01226	0.00888	1	1	1	3
São Paulo x Florianópolis	0.53968	284	0.00526	0.00284	1	1	1	3
^*^Paraná x Florianópolis	0.07563	220	0.00412	0.00031	1	0	0	3
São Paulo x ^*^Paraná	0.00000	68	0.00174	0.00000	0	0	2	1

Km: geographic distance between localities (in km); *F*_*ST*_: pair-wise estimates of population differentiation; *D*_*XY*_: average number of nucleotide substitutions per site between populations; *D*_*a*_: number of net nucleotide substitutions per site between populations; *S*_*S*_: number of shared polymorphisms between the two populations; *S*_*F*_: number of fixed differences between the two populations; *S*_*1*_: polymorphisms found only in the first population from the first column; *S*_*2*_: polymorphisms found only in the second population from the first column. ^*^Paraná: since only one sequence is available from Antonina (PR), it was combined with the sequences from Ilha do Mel (PR) under the label ‘Paraná’ to simplify the analysis, resulting in a total of four sequences for ^*^Paraná. Samples from Trinidad and São Paulo State were obtained from GenBank (accession numbers: OQ272307 - OQ272313, OQ272315 - OQ272319, and KU551287).

The highest differentiation (*F*_*ST*_ range: 0.91 - 0.98) was found between Trinidad and the other populations, along with a high number of fixed differences (range: 10 - 22) and no shared polymorphisms. These results align with Carvalho-Pinto’s isoenzyme analysis,^10^ which showed that the *An. bellator* population from Trinidad is genetically distinct from Brazilian populations, suggesting an early stage of speciation.

A high level of differentiation (*F*_*ST*_ range: 0.90 - 0.96) was also observed in comparisons involving Camacan and the other populations. These also showed a significant number of fixed differences (range: 9 - 12). These results align with those of Voges et al.,^11^ who reported *F*_*ST*_ > 0.6 for the *timeless* and *Clock* genes. According to Hey & Pinho,^25^ *F*_*ST*_ values above 0.35 indicate distinct species, while lower values suggest populations within the same species. For example, the comparison between Florianópolis and ^*^Paraná populations shows a low *F*_*ST*_ value (*F*_*ST*_ = 0.075), and no fixed differences, suggesting they belong to the same species, which may be due to their close geographical proximity (220 km). However, despite a similarly short distance (280 km), the Bahia populations - Camacan and Itaparica - have a high *F*_*ST*_ value (> 0.9), suggesting they belong to different species.

The Itaparica population also exhibits high *F*_*ST*_ values (> 0.7) when compared to southern and southeastern populations. These results align with the isoenzyme analysis by Carvalho-Pinto & Lourenço-de-Oliveira,^10^ which shows that southern populations are genetically close but distant from Itaparica.

Among the southern populations, the highest *F*_*ST*_ values (> 0.7) were observed in comparisons involving Ilha Grande (RJ). This result was unexpected, as Voges et al.^11^ reported much lower *F*_*ST*_ values (< 0.20) for these same comparisons using the *timeless* and *Clock* nuclear genes. The difference in *F*_*ST*_ values between the mitochondrial *COI* gene and the nuclear genes *timeless* and *Clock* may be due to mutation rates. Mitochondrial DNA generally has a higher mutation rate than nuclear DNA in animals.^26^ ^,^ ^27^ As a result, mutations are likely to occur first in mitochondrial regions, such as the *COI* gene, especially in cases of reproductive isolation. This makes *COI* a useful marker for distinguishing closely related species. It has been shown that mitochondrial genes tend to reveal more phylogenetic groups than nuclear genes in mosquitoes.^28^

The *F*_*ST*_ results align with the phylogenetic tree and haplotype network presented earlier, indicating that the analysed *An. bellator* populations represent at least three incipient species: Trinidad, *An. bellator* A and *An. bellator* B. Trinidad is genetically distinct from all Brazilian populations and from Colombia, supporting findings from a previous isoenzyme study.^10^ *An. bellator* B is composed exclusively of sequences from Camacan, consistent with Voges et al.^11^ *An. bellator* A includes populations from Itaparica, other southern regions of Brazil, and Colombia. Notably, preliminary findings suggest the existence of two distinct incipient species within *An. bellator* A: (i) the population from Itaparica, aligning with Carvalho-Pinto & Lourenço-de-Oliveira,^10^ and (ii) the population from Ilha Grande, which represents a novel finding from this study.

This study is the first to jointly analyse *An. bellator* samples from Colombia, Trinidad, Itaparica, Camacan, and southern Brazilian regions, revealing that the *An. bellator* population from Trinidad is genetically distinct from all Brazilian populations and from Colombia. This analysis also revealed that the Camacan population forms a distinct group from those in Itaparica, with both differing from the southern Brazilian populations, yet none of these groups can be distinguished morphologically using current taxonomic keys.

Furthermore, previous studies have shown a consistent pattern of separation between the northern and southern segments of the Atlantic Forest, observed across several fauna (including *An. cruzii* s.s.) and flora groups. This divergence typically occurs around northern Espírito Santo and southern Bahia, suggesting a shared vicariant event.^29^ ^,^ ^30^

*In conclusion* - This is the first time that *An. bellator* populations from Itaparica, Camacan, and the southern Brazilian regions have been analysed together, revealing that *An. bellator* from Camacan forms a distinct group from those in Itaparica, with both differing from the southern populations. Additionally, the data suggests that the southern populations may represent different incipient species, particularly in the case of Ilha Grande. This analysis also revealed that the *An. bellator* population from Trinidad is genetically distinct from all Brazilian populations and from Colombia. This divergence is strongly supported by (i) fixed differences, (ii) high *F*_*ST*_ values, (iii) *COI* tree topology with strong statistical support, and (iv) the haplotype network.

Further research is needed to better understand the genetic structure of *An. bellator* in the Atlantic Forest, and the use of additional molecular markers, along with intermediate populations, may provide valuable insights.
