# Supplementary material for: Population structure of Anopheles (Kerteszia) bellator in the Brazilian Atlantic Forest
Source: Mem Inst Oswaldo Cruz. 2025 Jul 21;120:e240287. doi: 10.1590/0074-02760240287 (PMC12286609; doi:10.1590/0074-02760240287)
Supplement: Supplementary file 1 [file 1678-8060-mioc-120-e240287-s.pdf]

TABLE I

Details of *Anopheles bellator* field collections

Sample ID	COI Fragment Length (pb)	Accession Number	Locality	State	Capture method	Latitude	Longitude	Sex	Year	Source
CAM01	514	PP422263	Camacan	Bahia	CDC Trap	-15.39191667	-39.86516667	F	2021	Collected for this study
CAM02	531	PP422264	Camacan	Bahia	CDC Trap	-15.39191667	-39.86516667	F	2021	Collected for this study
CAM03	466	PP422265	Camacan	Bahia	CDC Trap	-15.39191667	-39.86516667	F	2021	Collected for this study
ABBA03	357	PP422261	Camacan	Bahia	CDC Trap	-15.39191667	-39.86516667	F	2015	(11)
ABBA04	420	PP422262	Camacan	Bahia	CDC Trap	-15.39191667	-39.86516667	F	2015	(11)
FLO01	678	PP422291	Ilha do Arvoredo, Florianópolis	Santa Catarina	CDC Trap	-27.29916667	-48.35638889	F	2022	Collected for this study
FLO02	680	PP422292	Ilha do Arvoredo, Florianópolis	Santa Catarina	CDC Trap	-27.29916667	-48.35638889	F	2022	Collected for this study
FLO03	521	PP422293	Ilha do Arvoredo, Florianópolis	Santa Catarina	CDC Trap	-27.29916667	-48.35638889	F	2022	Collected for this study
FLO04	500	PP422294	Ilha do Arvoredo, Florianópolis	Santa Catarina	CDC Trap	-27.29916667	-48.35638889	F	2022	Collected for this study
FLO05	516	PP422295	Ilha do Arvoredo, Florianópolis	Santa Catarina	CDC Trap	-27.29916667	-48.35638889	F	2022	Collected for this study
FLO06	453	PP422296	Ilha do Arvoredo, Florianópolis	Santa Catarina	CDC Trap	-27.29916667	-48.35638889	F	2022	Collected for this study
FLO07	496	PP422297	Ilha do Arvoredo, Florianópolis	Santa Catarina	CDC Trap	-27.29916667	-48.35638889	F	2022	Collected for this study
FLO08	609	PP422298	Ilha do Arvoredo, Florianópolis	Santa Catarina	CDC Trap	-27.29916667	-48.35638889	F	2022	Collected for this study
ABIM01	426	PP422287	Paranaguá, Ilha do Mel	Paraná	Bromeliaceae water	-25.516167	-48.333972	F	2014	(11)
ABIM05	649	PP422288	Paranaguá, Ilha do Mel	Paraná	Bromeliaceae water	-25.516167	-48.333972	F	2014	(11)
ABIM07	679	PP422289	Paranaguá, Ilha do Mel	Paraná	Bromeliaceae water	-25.516167	-48.333972	F	2014	(11)
ANT01	303	PP422290	Antonina	Paraná	Unknown	-25.42861111	-48.71194444	F	Unknown	Collected for this study
ILH01	661	PP422276	Sítio Forte, Ilha Grande, Angra dos Reis	Rio de Janeiro	Bromeliaceae water	-23.11791667	-44.27497222	F	2019	Collected for this study
ILH02	685	PP422277	Sítio Forte, Ilha Grande, Angra dos Reis	Rio de Janeiro	Bromeliaceae water	-23.11791667	-44.27497222	F	2019	Collected for this study
ILH03	729	PP422278	Sítio Forte, Ilha Grande, Angra dos Reis	Rio de Janeiro	Bromeliaceae water	-23.11791667	-44.27497222	F	2019	Collected for this study
ILH04	638	PP422279	Sítio Forte, Ilha Grande, Angra dos Reis	Rio de Janeiro	Bromeliaceae water	-23.11791667	-44.27497222	F	2019	Collected for this study
ILH05	658	PP422280	Sítio Forte, Ilha Grande, Angra dos Reis	Rio de Janeiro	Bromeliaceae water	-23.11791667	-44.27497222	F	2019	Collected for this study
ILH06	631	PP422281	Sítio Forte, Ilha Grande, Angra dos Reis	Rio de Janeiro	Bromeliaceae water	-23.11791667	-44.27497222	M	2019	Collected for this study
ILH07	505	PP422282	Sítio Forte, Ilha Grande, Angra dos Reis	Rio de Janeiro	Bromeliaceae water	-23.11791667	-44.27497222	F	2019	Collected for this study
ILH08	468	PP422283	Sítio Forte, Ilha Grande, Angra dos Reis	Rio de Janeiro	Bromeliaceae water	-23.11791667	-44.27497222	F	2019	Collected for this study
ABSF22	294	PP422286	Sítio Forte, Ilha Grande, Angra dos Reis	Rio de Janeiro	Bromeliaceae water	-23.11791667	-44.27497222	F	2012	(11)
ABSF60	467	PP422284	Sítio Forte, Ilha Grande, Angra dos Reis	Rio de Janeiro	Bromeliaceae water	-23.11791667	-44.27497222	F	2012	(11)
ABSF61	467	PP422285	Sítio Forte, Ilha Grande, Angra dos Reis	Rio de Janeiro	Bromeliaceae water	-23.11791667	-44.27497222	F	2012	(11)
ITA01	618	PP422266	Itaparica	Bahia	Bromeliaceae water	-13.06002778	-38.77641667	F	2019	Collected for this study
ITA02	675	PP422267	Itaparica	Bahia	Bromeliaceae water	-13.06002778	-38.77641667	F	2019	Collected for this study

Sample ID	COI Fragment Length (pb)	Accession Number	Locality	State	Capture method	Latitude	Longitude	Sex	Year	Source
ITA03	721	PP422268	Itaparica	Bahia	Bromeliaceae water	-13.06002778	-38.77641667	F	2019	Collected for this study
ITA04	673	PP422269	Itaparica	Bahia	Bromeliaceae water	-13.06002778	-38.77641667	F	2019	Collected for this study
ITA05	602	PP422270	Itaparica	Bahia	Bromeliaceae water	-13.06002778	-38.77641667	F	2019	Collected for this study
ITA06	611	PP422271	Itaparica	Bahia	Bromeliaceae water	-13.06002778	-38.77641667	F	2019	Collected for this study
ITA07	725	PP422272	Itaparica	Bahia	Bromeliaceae water	-13.06002778	-38.77641667	F	2019	Collected for this study
ITA08	723	PP422273	Itaparica	Bahia	Bromeliaceae water	-13.06002778	-38.77641667	F	2019	Collected for this study
ITA09	708	PP422274	Itaparica	Bahia	Bromeliaceae water	-13.06002778	-38.77641667	F	2019	Collected for this study
ITA10	739	PP422275	Itaparica	Bahia	Bromeliaceae water	-13.06002778	-38.77641667	F	2019	Collected for this study

This file provides comprehensive information about each sample generated in this study. Capture method: Bromeliaceae water - immatures collected from bromeliad water and reared in the laboratory; CDC trap: adults collected using CDC traps; Sex: sex of each sample - M for males, F for females. Source: Voges et al.[11] - DNA kindly donated by Voges et al.[11]; or samples collected specifically for this study.

TABLE II

Polymorphisms of *Anopheles bellator* populations

Populations	n	*S*	θ	π	D_T
Trinidad	03	00	0.0000	0.0000	-
Itaparica (BA)	10	11	0.0071	0.0094	1.4593
Camacan (BA)	05	03	0.0060	0.0059	-0.1747
Ilha Grande (RJ)	11	02	0.0023	0.0017	-0.7781
São Paulo	10	04	0.0024	0.0013	-1.6670
*Paraná	04	01	0.0021	0.0019	-0.6123
Florianópolis (SC)	08	04	0.0034	0.0032	-0.2217

n: number of DNA sequences per population; *S*: number of polymorphic (segregating) sites; θ: nucleotide diversity based on the total number of mutations (Eta); π: nucleotide diversity based on the average number of pair-wise differences; D_T: Tajima's D test[20]; *Paraná: since only one sequence is available from Antonina (PR), it was combined with the sequences from Ilha do Mel (PR) under the label 'Paraná' to simplify the analysis, resulting in a total of four sequences for *Paraná. The samples from Trinidad and São Paulo State were obtained from GenBank (accession numbers: OQ272307 - OQ272313, OQ272315 - OQ272319, and KU551287). Tajima's D test could not be calculated for the Trinidad population because a minimum of four sequences is required.
